# Daclatasvir-based regimens in HCV cirrhosis: experience from the Italian early access program

**DOI:** 10.1038/s41598-018-36734-0

**Published:** 2019-01-24

**Authors:** Vincenza Calvaruso, Chiara Mazzarelli, Laura Milazzo, Lorenzo Badia, Luisa Pasulo, Giovanni Guaraldi, Raffaella Lionetti, Erica Villa, Vanni Borghi, Paola Carrai, Alfredo Alberti, Marco Biolato, Guido Piai, Marcello Persico, Teresa Santantonio, Martina Felder, Mario Angelico, Marzia Montalbano, Rossella Letizia Mancusi, Antonio Grieco, Elena Angeli, Gianpiero D’Offizi, Stefano Fagiuoli, Luca Belli, Gabriella Verucchi, Massimo Puoti, Antonio Craxì

**Affiliations:** 1 Gastroenterology and Hepatology Unit, Dipartimento Biomedico di Medicina Interna e Specialistica, University of Palermo, Palermo, Italy; 2 Gastroenterology and Liver unit, Niguarda Ca’ granda, Milan, Italy; 3 Section of Infectious Diseases, L. Sacco University Hospital, Milan, Italy; 4 Infectious Diseases Unit - Research Centre for the Study of Hepatitis, Department of Medical and Surgical Sciences (DIMEC), University of Bologna, Bologna, Italy; 5 Gastroenterology and Liver unit, San giovanni XXIII Hospital, Bergamo, Italy; 6 Infectious Disease Unit - Azienda Ospedaliero Universitaria di Modena, Modena, Italy; 7 Ist. Naz. Malattie Infettive L. Spallanzani, Rome, Italy; 8 Gastroenterology Unit Azienda Ospedaliero Universitaria di Modena, Modena, Italy; 9 Liver transplantation Unit, Pisa, Italy; 10 Department of Molecular Medicine, University of Padova, Padova, Italy; 11 Liver Transplant Medicine, Fondazione Policlinico Universitario A. Gemelli IRCCS, Università Cattolica del Sacro Cuore, Roma, Italy; 12 Azienda Ospedaliera San Sebastiano-Caserta, Caserta, Italy; 13 Salerno University of Medicine, Fisciano, Italy; 14 Clinic of Infectious Diseases, University of Foggia, Foggia, Italy; 15 Gastroenterology Unit, Ospedale Centrale Bolzano, Bolzano, Italy; 16 Hepatology Unit, Tor vergata university, Rome, Italy; 17 C.R.E.A. Sanità - University of Tor Vergata, Rome, Italy; 18 Section of Infectious Diseases, Niguarda Ca’ granda, Milan, Italy

## Abstract

We reported the efficacy and safety data for daclatasvir (DCV)-based all-oral antiviral therapy in patients treated in the Italian compassionate-use program. 275 patients were included (202 male-73.5%, mean age: 57.4 years, 62 HIV-coinfected, 94 with recurrence of hepatitis C post-OLT). Forty-nine patients (17.8%) had Child-Pugh B, Genotype(G) distribution was: G1a:72 patients (26.2%), G1b:137 (49.8%); G3:40 (14.5%) and G4:26 (9.5%). Patients received DCV with sofosbuvir(SOF) (n = 221, 129 with ribavirin(RBV) or with simeprevir (SMV) or asunaprevir (ASU) (n = 54, 19 with RBV) for up to 24 weeks. Logistic regression was used to identify baseline characteristics associated with sustained virological response at week 12 post-treatment (SVR12). Liver function changes between baseline and follow up were assessed in 228 patients. 240 patients achieved SVR12 (87.3%), post transplant and HIV co-infected patients were equally distributed among SVR and no SVR (35% vs 34.3%; p = 0.56 and 24.2% vs 11.4%, p = 0.13, respectively). SVR rate was significantly higher with the combination DCV + SOF compared with DCV + SIM or ASU (93.2% vs 63.0%, p < 0.0001). Bilirubin value (OR: 0.69, CI95%: 0.54–0.87, p = 0.002) and regimen containing SOF (OR: 9.99, CI95%: 4.09–24.40; p < 0.001) were independently related with SVR. Mean albumin and bilirubin values significantly improved between baseline and follow-up week 12. DCV-based antiviral therapy was well tolerated and resulted in a high SVR when combined with SOF either in pre-transplant and in OLT patients and in “difficult to treat” HCV genotypes. Regimens containing DCV in combination with NS3 protease inhibitors obtained suboptimal results.

## Introduction

The prevalence of cirrhosis and the incidence of its complications increased in the last years in several countries, related to the long history of infection among subjects with chronic hepatitis C (CHC)^1,2^. The natural history of HCV-related cirrhosis is characterized by the consequences of portal hypertension and by hepatocellular carcinoma (HCC). Early identification and treatment of HCV patients with advanced liver disease can reduce the rate of decompensation and the risk of HCC^3–5^. During the last years, different interferon-(IFN) free regimens have been approved for use in CHC. The safety and efficacy of the combination of direct-acting antivirals (DAAs), may allow to treat patients in whom the IFN containing regimens had a very limited efficacy and tolerance or to achieve the SVR in patients who failed a previous IFN based antiviral treatment. Daclatasvir (DCV) is a potent pangenotypic NS5A inhibitor that has been shown to be safe and effective for the treatment of patients CHC by different HCV genotypes, including those with cirrhosis^6^. Data from phase III study showed an high proportion of SVR in patients with cirrhosis also in genotype 3 infection^7^. Moreover, DCV has been shown to be safe in decompensated cirrhosis^8^.

Compassionate use programs for DAAs established by Bristol Myers Squibb (BMS) provided access to drugs that have yet to be approved for high-priority patients with advanced cirrhosis who are at high risk of decompensation and/or death in a short time span.

The available data have shown that the efficacy and safety of DCV-based treatments in patients with cirrhosis are not significantly different from those obtained in non cirrhotic subjects, however there are limited data on IFN-free regimens in advanced liver disease, and the impact of HCV clearance on patients with decompensated cirrhosis as well as on patients awaiting liver transplantation needs further evaluation.

## Aims

The main proposals of the study were:

- To evaluate the efficacy and safety of DCV-based IFN-free regimens in patients with HCV cirrhosis treated through the Patient Supply Program in an Italian real-life clinical setting.
- To assess factors associated with failure of interferon-free DCV based antiviral therapy.
- To assess the therapeutic efficacy as measured by changes in liver function tests during treatment and follow up,

## Patients and Methods

The BMS compassionate expanded access program (EAP) provided treatment access for patients with HCV who had liver cirrhosis at high risk of decompensation and mortality.

The EAP was initiated by BMS on March, 2013.

Recruitment was stopped on may, 2015, when DCV received marketing approval by Agenzia Italiana del Farmaco (AIFA). Patients were recruited at 31 sites in Italy via a network. The data analysis cut-off was January 30, 2017.

Patients could be treatment naive or treatment experienced to an interferon (IFN)-based therapy, with or without ribavirin (either non responder or relapse, or IFN-intolerant.

Patients could be previously transplanted with an evidence of severe recurrence of HCV infection after liver transplantation.

Patients could be HIV-infected with documented HIV RNA suppression due to regular antiretroviral treatment.

All patients included in the compassionate use have been treated with DCV combined with Asunaprevir (ASU - NS3 protease inhibitor)^9^ or with Simeprevir (SIM - NS3/4 A protease inhibitor) or with Sofosbuvir (SOF - NS5B nucleotide polymerase inhibitor). The type of regimens as well as the use of ribavirin was totally related to physician’s discretion.

The study was done in accordance with the International Conference on Harmonisation guidelines and the principles of the Declaration of Helsinki. Ethical Committee of Azienda Ospedaliera Universitaria Policlinico P. Giaccone of Palermo (Comitato Etico I) approved the collection and analysis of the data. Written informed consent was provided by any patient enrolled in the study before any study-specific procedures were carried out.

### Efficacy and safety assessments

“Efficacy and safety data were collected according to good clinical practice and Italian laws on drug safety and pharmacovigilance”. HCV RNA testing was performed by the local laboratory at each site. HCV RNA levels were quantified using the Abbott Real Time HCV PCR assay (lower limit of quantification,12 IU/ml, Abbott Diagnostics, USA) or Real time PCR (Roche). (Range:15–100.000.000 IU/ml).

Laboratory assessments including international normalised ratio (INR), albumin concentration, bilirubin concentration, alanine aminotransferase (ALT) levels, and hemoglobin concentration, were performed at baseline, every 4 weeks during treatment thereafter and at week 12 after treatment suspension.

Plasma HCV RNA concentration has been assessed at baseline, week 4 of treatment, end of treatment, and week 12 after treatment.

Data on adverse events has been recorded throughout the study.

Patient visits and follow-up, as well as laboratory testing, were done according to the sites’ local standards.

Laboratory and safety data were reported on an ongoing basis in a spreadsheet to the principal investigator.

### Outcomes

The primary efficacy outcome was the rate of patients achieving SVR (undetectable HCV RNA) 12 weeks after the end of therapy.

Viral breakthrough and relapse were defined as detectable plasma HCV RNA levels after achieving undetectability, during treatment and after the end of treatment, respectively.

Secondary endpoints included effect of antiviral therapy on markers of liver function, such as INR, albumin levels, and bilirubin levels, CPT, MELD score; safety events consistent with liver adverse events and deaths during the study.

### Statistical analysis

A descriptive analysis is provided for socio-demographic parameters on enrolment, as well as clinical variables related to liver function. Mean, standard deviation, range, median, interquartile range, number of missing data, and 95% confidence intervals were determined for continuous variables; absolute frequency, 95% confidence intervals and number of missing data for categorical variables. Associations between qualitative variables were measured with a χ2 test, while differences between quantitative variables were evaluated with a Student’s t test. Logistic regression univariate and multivariate analysis was performed to assess the predictors of SVR. Statistical computations were performed with the SPSS® Statistics package version 20.0.0. Data were analysed according to intention to treat (ITT).

## Results

**Figure 1:**
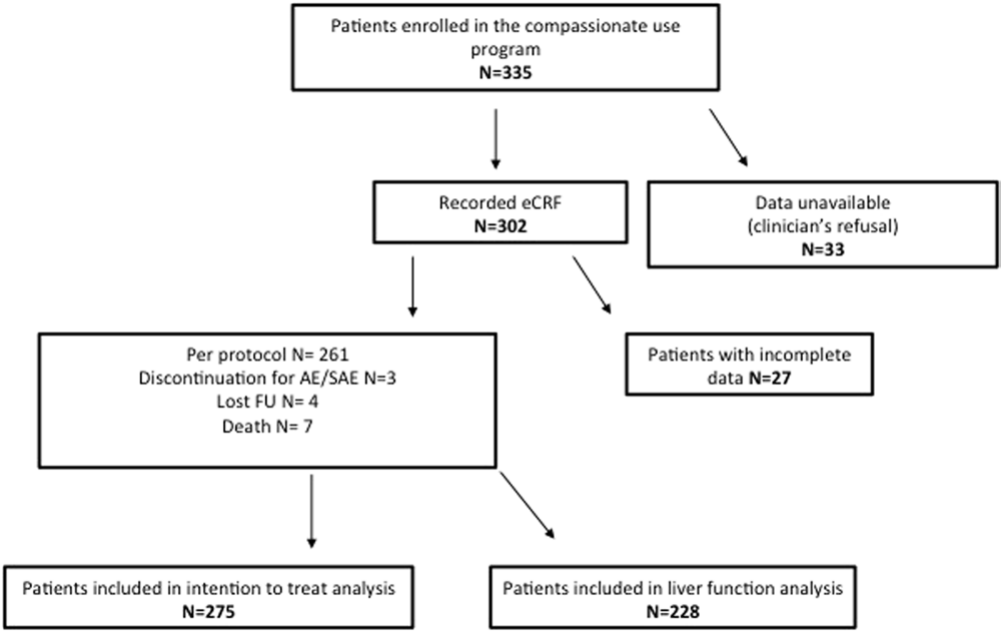
Flow chart of Daclatasvir-based Compassionate use program.

DCV EAP provided treatment access for 335 patients HCV-infected patients with cirrhosis. In 33 cases, clinicians refused to provide data and in other 27 data were incomplete, therefore 60 patients were excluded from the analysis. Two-hundreds-seventyfive patients were included in the ITT population and 228 were also evaluated for the improvement of liver function tests (Fig. 1).

Baseline demographics and disease characteristics are presented in Table 1. Forty-nine patients (22.5%) with Child-Pugh class B cirrhosis were included. HIV coinfection was present in 62 cases (22.6%) and 94 patients had a recurrence after liver transplantation (34.2%).

**Table 1:** Baseline characteristics of 275 patients with HCV cirrhosis treated with DCV based antiviral treatment.

Variable	N = 275
Age – yrs mean ± SD (median)	57.4 ± 8.6 (55)
Male gender (%)	202 (73.5)
Creatinine – mg/dl	1.01 ± 0.8
ALT – IU/L	70.0 ± 58.7
Total bilirubin – mg/dl	1.8 ± 1.5
INR- %	1.2 ± 0.3
Albumin – g/dl	3.7 ± 0.6
Platelets – × 10^3^/mL	92.5 ± 51.4
MELD	11.5 ± 4.4 (10.4)
Child Pugh B*	49 (22.5)*
Genotype 1a	72 (26.2)
1b	137(49.8)
3	40 (14.5)
4	26 (9.5)
HCV RNA – IU/ml	2,628,888.7 ± 8,199,742.5
HCVRNA > 1.000.000 IU/mL	39.60%
Naïve to Antiviral Therapy (%)	62 (22.6)
HIV coinfection (%)	62 (22.7)
Post-OLT	94 (34.2)

Data available on 217 pati.

The combination of DCV and SOF was used in 221 (80.4%) patients (129 with RBV and 92 without RBV) whereas DCV was associated with protease inhibitors in 54 (19.6%) patients, SIM in 36 cases (16 with RBV and 20 without RBV) and ASU in 18 cases (3 with RBV and 15 without RBV).

The antiviral regimens used according to HCV genotype are shown in Supplementary Table.

The combination of DCV with protease inhibitors was used in 13 patients (50%) of patients with HCV genotype 4 and in 41 (29.9%) of patients with genotype 1b, All patients with HCV genotype 1a and 3 received DCV plus SOF.

By ITT analysis, overall, 240 patients (87.3%) obtained SVR12. Nine patients relapsed (3.3%), twelve patients experienced a breakthrough (4.4%) three patients discontinued therapy for adverse events (1.1%) and eleven were lost or died between the end of treatment and the week 12 of follow up (4%). By univariate analysis, the proportion of patients achieving SVR12 did not differ substantially by age, gender, renal function, MELD and Child-Pugh score, baseline HCV RNA concentrations, previous antiviral treatment, HIV coinfection and OLT status (Table 2). Conversely the mean value of total bilirubin was higher in the group that did not achieve SVR, compared with the SVR12 one (2.2 ± 1.5 mg/dl vs 1.7 ± 1.5 mg/dl; p = 0·07); The rates of SVR in patients with basal bilirubin value < or ≥2 mg/dl were 96.5% and 87.2% (p = 0.012) respectively.

**Table 2:** Clinical features according to SVR 12 in 275 patients with HCV cirrhosis treated with DCV based antiviral treatment.

Variable	SVR (n = 240)	No SVR (n = 35)	p value	Multivariate analysis Adjusted OR(95%CI) p value
Age – yrs mean ± SD	57.5 ± 8.2	56.9 ± 10.8	0.68	—
Male gender (%)	178 (74.2)	24 (68.6)	0.3	—
Creatinine – mg/dl	1.03 ± 0.9	0.9 ± 0.3	0.34	—
ALT – IU	71.0 ± 58.3	62.6 ± 61.9	0.43	—
Total bilirubin – mg/dl	1.7 ± 1.5	2.2 ± 1.5	0.07	0.69 (0.54–0.87) 0.002
INR- %	1.2 ± 0.3	1.3 ± 0.4	0.23	—
Albumin – g/dl	3.7 ± 0.6	3.6 ± 0.6	0.26	—
Platelets - × 10^3^/mL	93,467 ± 53,415	85,387 ± 33,687	0.41	—
MELD	11.4 ± 4.2	12.2 (5.8)	0.45	—
Child Pugh B*	45 (23.1)*	4 (18.2)	0.6	—
Genotype 1a	66 (27.5)	6 (17.1)	0.006#	1.92 (0.67–5.50) 0.22
1b	119(49.6)	18 (51.4)		
3	37 (15.4)	3 (8.6)		
4	18 (7.5)	8 (22.9)		
HCVRNA > 1,000,000 IU/ml	95 (39.6)	11 (31.4)	0.28	—
Naïve to Antiviral Therapy (%)	53 (22.1)	9 (25.7)	0.67	—
HIV coinfection (%)	58 (24.2)	4 (11.4)	0.13	—
Post-OLT	82 (35.0)	12 (34.3)	0.56	—
DAC/SOF ± RBV	206 (85.8)	15 (42.9)	<0.001	9.99 (4.09–24.40) < 0.001
DAC/SIM-ASU ± RBV	34 (14.2)	20 (57.1)		
Ribavirin: no	107 (44.6)	20 (57.1)	0.113	
yes	133 (55.4)	15 (42.9)		

*Data available on 217 patients.

**Figure 2:**
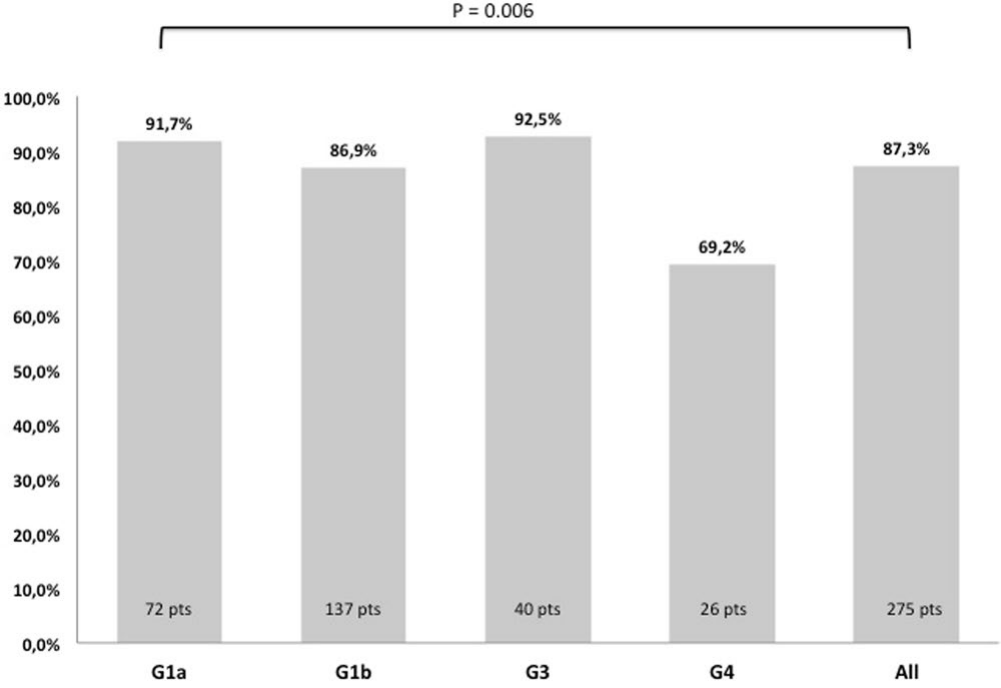
Sustained virological response at post-treatment week 12 (SVR12) according to Daclatasvir based regimen.

The difference in the proportion of patients achieving SVR12 by HCV genotype was significant (p = 0.006) as shown in Table 2 and Fig. 2. SVR12 rates were 91.7%, 86.9%, 92.5% and 69.2% in patients with genotype 1a, 1b, 3 and 4 respectively.

**Figure 3:**
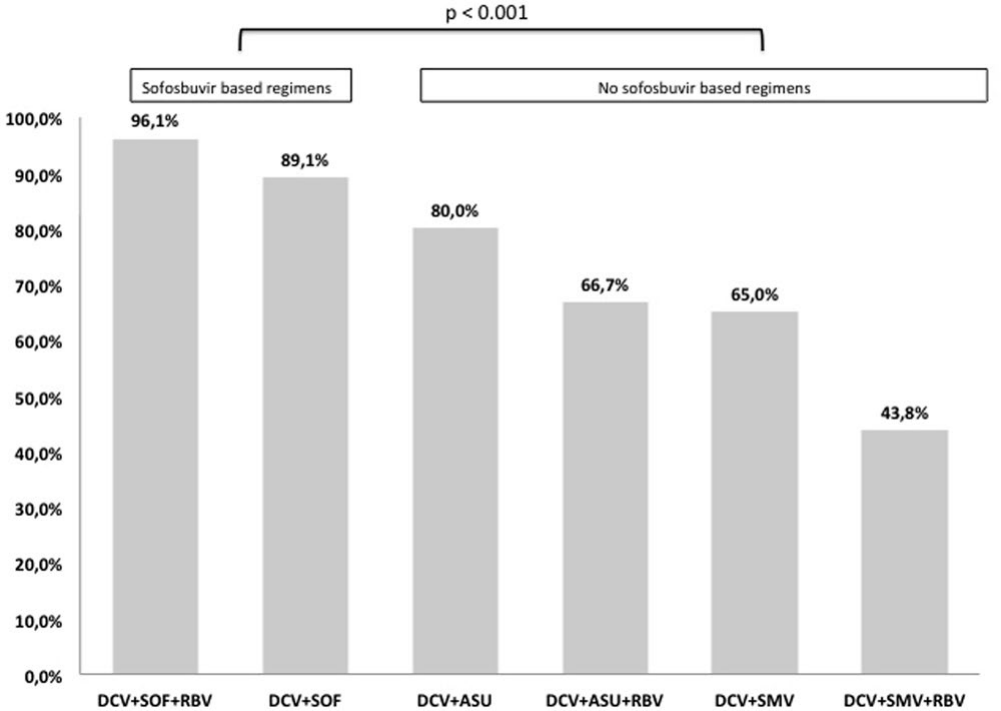
Modification of bilirubin, albumin and MELD values after therapy.

Finally, the rate of SVR12 was significantly higher with the association of DCV plus SOF than DCV plus protease inhibitors (93.2% vs 63%, p < 0.001; Fig. 3).

The rate of genotype 4 patients who received DCV combined with protease inhibitors was significantly higher respect than patients infected with other HCV genotypes (50% vs 16.5%, p < 0.001).

No relationship was found between the use of RBV and the rate of SVR. (55.4% in SVR vs 42.9%, in no SVR; p = 0.113).

By multivariate analysis, bilirubin value (OR: 0.69, CI95%: 0.54–0.87, p = 0.002) and the combination of DCV with SOF (OR: 9.99; CI: 4.09–24.40, <0.001) were independently related with SVR12. Table 2.

### Safety analysis

Twenty-five of 275 patients (9.1%) had an adverse event. Five patients developed mild infectious disease (1.8%) as well as 4 patients (1.4%) presented at least an episode of hepatic decompensation (2 ascites and 2 hepatic encephalopaty).

Severe anaemia hemoglobin (<8 g/dL) occurred in four patients (1.4%) who received blood transfusions. Jaundice (with bilirubin value higher than 10 mg/dl) occurred in three patients (1.1%). Other adverse events recorded were: cheilitis in three (1.1%), asthenia in two (0.7%), headache in two (0.7%) hyper eosinophilia and insomnia in one each.

Only three (1.1%) of 275 patients discontinued study drugs due to an adverse event.

Seven patients (2.5%) died during the study, three of liver failure, and four as results of HCC progression, OLT complications, gastric bleeding and stroke respectively.

### Liver function tests

Changes of liver and renal function parameters throughout the study period are shown in Table 3. Between baseline and the end of therapy we observed a significant improvement in serum albumin levels (3.7 ± 0.6 vs 3.9 ± 0.6 g/dl; p value < 0.001) while serum creatinine levels significantly increased (p = 0.013). No changes were seen in total bilirubin, INR and MELD score. A significant improvement of albumin was confirmed at week 12 of follow-up (p < 0.001), whereas no significant change from baseline was observed in creatinine levels. However, total bilirubin values significantly decreased from baseline to the end of follow-up (p < 0.001).

**Table 3:** Liver and renal function parameters change from Baseline to End of therapy and to Follow-up week 12.

	Baseline value	End of therapy value Follow up week 12 value	P value
Albumin - g/dl (mean ± SD)	3.7 ± 0.6	3.9 ± 0.6	**<0.001** <**0.001**
		4.0 ± 0.6	
Bilirubin - mg/dl (mean ± SD)	1.8 ± 1.5	1.7 ± 2.7	0.53 <**0.001**
		1.3 ± 1.1	
INR (mean ± SD)	1.2 ± 0.3	1.2 ± 0.3	0.65
		1.2 ± 0.2	0.27
Creatinine - mg/dl (mean ± SD)	1.0 ± 0.9	1.4 ± 1.1	**0.013**
		1.1 ± 0.9	0.33
MELD value (mean ± SD)	11.6 ± 4.4	11.3 ± 4.4	0.16
		10.8 ± 4.7	0.08

MELD value decreased, although not significantly, between baseline and follow-up week 12. An improvement of MELD score was observed in 111 patients (48.7%) (Supplementary Figure).

## Discussion

Overall, the antiviral therapy with DCV based regimens, with or without ribavirin in patients with cirrhosis in a real life setting, resulted in a high proportion of patients achieving SVR12 and in a low rate of side effects.

The antiviral drugs, provided within a EAP, allowed the clinicians to treat a large number of patients with advanced liver disease at high risk of decompensation and mortality and to collect data on efficacy and safety of the different regimens containing DCV on difficult to treat population that is often excluded from clinical trials. SVR rate observed in our cohort was 87.3% with SVR12 higher than 90% in patient treated with SOF and DCV also in the subgroups more difficult to cure, such as decompensated cirrhosis, genotype 1a or 3 and/or HIV co-infection.

Similarly to the what reported in phase III clinical trials (ALLY program), the combinations of DCV/SOF is highly effective in eradicating HCV even in patients with advanced liver disease^7,10–12^.

By contrast, the combination of DCV with protease inhibitors (ASU/SIM) showed a suboptimal efficacy when compared to the available data from clinical trials in patients with a less advanced disease^13,14^. Indeed, in our study, cirrhotic patients treated with combination of DCV and SIM or ASU achieved SVR12 in less than 70% of cases and also the lower SVR rate observed in genotype 4 patients can be related to the high percentage of patients treated with DCV combined with protease inhibitors in this subgroup.

This is the first real life study that compared in a consistent number of patients (221 treated with DCV/SOF and 54 treated with DCV/SIM or ASU) the efficacy of different DCV-based regimens in patients with cirrhosis and/or HIV coinfection. In fact, previously, only Fontana *et al*.^15^ compared, in a cohort of post-transplanted HCV patients, the combination of DCV/SOF and DCV/PI and similarly to our results they demonstrated a SVR rates of 91% and 72% in patients treated with DCV/SOF and DCV/SIM respectively.

Ishigami *et al*.^16^ have reported that patients with advanced fibrosis presented a lower rate of SVR achieving only 90% of SVR compared to 95% of patients with mild liver disease. However, in this study severe fibrosis was defined with a value of FIB 4 index > 3.25 and not decompensated liver patients were enrolled in this study,

According to the results of other community based expanded access programs^17^ with similar high proportion of patients with Child-Pugh B, we have confirmed the high SVR rates obtained by the combination of DCV/SOF regardless the use of RBV, the HIV coinfection and the transplant condition. HCV genotype was associated with SVR at univariate analysis with significant difference of SVR rate between genotype 1a, 1b and 3 versus genotype 4. The highest rate of SVR12 was observed among 40 genotype 3 patients (92.5%) followed by the 72 genotype 1a patients (91.7%). All of these patients received the combination of DCV/SOF associated in an higher percentage of cases with RBV. Conversely, the lowest SVR rate was observed among genotype 4 patients (69.2%) who received DCV associated with PI in 50% of cases. In order to better understand the reasons of suboptimal efficacy of DCV based regimens in genotype 4 patients it is worth taking into account also the results of Fourati *et al*.^18^ which, in a French cohort, have recently recorded an high rate of virological failure DAA-treated patients infected with genotype 4. Moreover, they have identified that the subtype 4r was significantly related with treatment failure due to the higher prevalence of S282C/T RASs development.

Together with the DCV regimen, also bilirubin value was independently associated with a lower response to antiviral therapy. This is consistent with previous studies which showed lower SVR12 rates in patients with advanced liver disease^12,16^ whose bilirubin is an indicator.

In order to assess the impact of SVR on the liver function of patients with advanced and decompensated liver disease we have evaluated biochemical changes between baseline and follow up in our cohort of patients. Results revealed a significant improvement of albumin and bilirubin values from baseline to follow-up week 12, along with a not significant decrease in mean MELD score.

This study has several limitations: retrospective study, no randomization of treatment allocation and the of use of RBV (used at physicians’ discretion), laboratory tests were conducted using the devices available at each centre and therefore, assay differences may have caused inconsistencies in laboratory-based efficacy and safety assessments. Furthermore, as a common limitation of real-life cohorts, the adverse events might be under-reported.

In spite of these limitations, this study reports data about a large cohort of patients with advanced liver disease treated with an oral DAA combination in a real-life setting.

In conclusion, data collected in this EAP demonstrated that DCV + SOF, with or without RBV in patients with advanced cirrhosis in a real-world setting is very effective, also for more “difficult to cure” HCV genotypes. The combination of DCV with prothease inhibitors (ASU/SIM confirmed its suboptimal efficacy and should be avoided in advanced liver disease. Treatment was well tolerated and was associated with improvements in liver function tests.

A longer follow-up period will allow the monitoring of clinical changes in these patients and will provide further insight into the long-term benefits of achieving SVR in patients with advanced liver disease.

## Electronic supplementary material

Supplementary figure

Supplementary table
