# Supplementary material for: Daclatasvir-based regimens in HCV cirrhosis: experience from the Italian early access program
Source: Sci Rep. 2019 Jan 24;9:585. doi: 10.1038/s41598-018-36734-0 (PMC6345835; doi:10.1038/s41598-018-36734-0)
Supplement: Supplementary file 2 — Supplementary table [file 41598_2018_36734_MOESM2_ESM.pdf]

Daclatasvir-based regimens in HCV cirrhosis: experience from the Italian early access program

AUTHORS:

Vincenza Calvaruso 1 (vincenza.calvaruso@unipa.it**), Chiara Mazzarelli 2 (**chiara.mazzarelli@ospedaleniguarda.it**), Laura Milazzo 3 (**laura.milazzo@unimi.it**), Lorenzo Badia 4 (**lorenzo.badia@aosp.bo.it**), Luisa Pasulo 5 (**lpasulo@ospedaliriuniti.bergamo.it**), Giovanni Guaraldi 6 (**giovanni.guaraldi@unimore.it**), Raffaella Lionetti 7 (**raffaella.lionetti@inmi.it**), Erica Villa 8 (**erica.villa@unimore.it**), Vanni Borghi 6 (**vanni.borghi@unimore.it**), Paola Carrai 9 (**p.carrai@ao-pisa.toscana.it**), Alfredo Alberti 10 (**alfredo.alberti@unipd.it**) , Marco Biolato 11 (**marco.biolato@policlinicogemelli.it), **Guido Piai 12 (**epatologia@gastrocaserta.it**), Marcello Persico 13 (**mpersico@unisa.it**), Teresa Santantonio 14 (**teresa.santantonio@unifg.it**), Martina Felder 15 (**MARTINA.FELDER@sabes.it**), Mario Angelico 16 (**angelico@med.uniroma2.it**), Marzia Montalbano 7,** (marzia.montalbano@inmi.it), **Rossella Letizia Mancusi 17**(rossella.letizia.mancusi@uniroma2.it), **Antonio Grieco 11** (antonio.grieco@Unicatt.it**), Elena Angeli 3 (**angeli.elena@hsacco.it**), Gianpiero D'Offizi 7 (**gianpiero.doffizi@inmi.it), **Stefano Fagiuoli 5 (**sfagiuoli@hpg23.it**), Luca Belli 2 (**luca.belli@ospedaleniguarda.it**), Gabriella Verucchi 4 (**gabriella.verucchi@unibo.it**), Massimo Puoti 18 (**massimopuoti@libero.it**), Antonio Craxì1 (**antonio.craxi@unipa.it**).**

Supplementary table . DCV based DAA regimen according HCV genotype

DAA regimen	G1a (72)	G1b (137)	G3 (40)	G4 (26)
DCV + SOF (92 pts)	30 (41.7)	44 (32.1)	10 (25)	8(30.8)
DCV + SOF + RBV (129)	42(58.3)	52 (38)	30 (75)	5 (19.2)
DCV + SMV (20 pts)	-	16 (11.7)	-	4 (15.4)
DVC + SIM + RBV (16)	-	8 (5.8)	-	8 (30.8)
DCV + ASU (15)	-	14 (10.2)	-	1 (3.8)
DCV + ASU + RBV (3)	-	3 (2.2)	-	-
